# Drivers and consequences of nest ectoparasite pressure in tit nestlings^☆^^☆☆^^☆☆☆^^☆☆☆☆^

**DOI:** 10.1016/j.ijppaw.2025.101075

**Published:** 2025-04-25

**Authors:** Sofía I. Arce, Jorge Garrido-Bautista, Catarina G. Cascão, Inês S.C. Vilhena, José Manuel Arjona, Ana Rita Cabral, Fábio Marengo, Joana Girão, Gregorio Moreno-Rueda, Jaime A. Ramos, Ana Cláudia Norte

**Affiliations:** a Laboratorio de Ecología de Enfermedades, Instituto de Ciencias Veterinarias del Litoral (ICiVet-Litoral), Universidad Nacional del Litoral (UNL) / Consejo Nacional de Investigaciones Científicas y Técnicas (CONICET), RP Kreder 2805, Esperanza, 3080, Santa Fe, Argentina; b Ludwig-Maximilians-Universität München, Biocenter, Großhaderner Str. 2, 82152, Planegg-Martinsried, Germany; c Department of Zoology, Faculty of Sciences, University of Granada. Avenida Fuente Nueva s/n, 18071, Granada, Spain; d University of Coimbra, MARE - Marine and Environmental Sciences Centre/ARNET, Department of Life Sciences, Calçada Martim de Freitas, Faculty of Sciences and Technology, Portugal; e Dipartimento di Scienze della Vita e Biologia dei Sistemi, Universitá Degli Studi di Torino, Italy

**Keywords:** Hole-nesting birds, Blood-sucking ectoparasites, Polychromasia, White blood cell count, Micronuclei

## Abstract

Ectoparasites impose significant costs to their hosts and modulate their life-history traits. We evaluated the prevalence and abundance of louse flies, blowflies, fleas and mites in great tits (*Parus major*) and blue tits (*Cyanistes caeruleus*) breeding in nest boxes in Central Portugal during two consecutive breeding seasons and assessed: (a) the potential physiological consequences of infestation for nestlings; (b) how nest box re-use and presence of anthropogenic materials in nests affected the ectoparasite abundance; (c) how host reproductive parameters were related to ectoparasitism; and (d) how different nest-dwelling arthropod groups, including ectoparasites, and their diversity correlated. Tit nestlings reared in nests with more blowflies showed symptoms of anaemia, such as lower haemoglobin levels and high erythrocyte maturation index, and tended to grow less. Nestlings from nests with higher number of obligatory parasitic mites had increased polychromasia, and blue tits tended to have lower probability to fledge. Great tit nestlings from nests with fleas also had increased polychromasia compared with those from non-infested nests. Nest box re-use increased the probability of infestation by louse flies and obligatory parasitic mites. In both tit species, broods that were reared later in the season had higher abundance of blowflies and obligatory parasitic mites in their nests. In great tit nests, anthropogenic materials were negatively correlated with flea abundance, and positively correlated with the abundance of Histeridae coleopterans. In great and blue tit nests, obligatory parasitic mites were less abundant when nests showed a higher abundance of Staphylinidae coleopterans and Collembola. Overall, this study shows strong negative effects of nest ectoparasite pressure, particularly blowflies and obligatory parasitic mites, on physiological and fitness measures of hole nesting birds.

## Highlights

• Mite infestation was higher in great tit nests than in blue tit nests.
• Infestation by mites and louse flies was linked to nest box re-use.
• Anthropogenic nest materials influenced the abundance of arthropods in the nest.
• Blowfly and obligatory parasitic mite infestation increased throughout breeding season.
• Blowflies and mites impacted nestling blood indices of respiratory capacity.

## Graphical abstract

**Figure:**
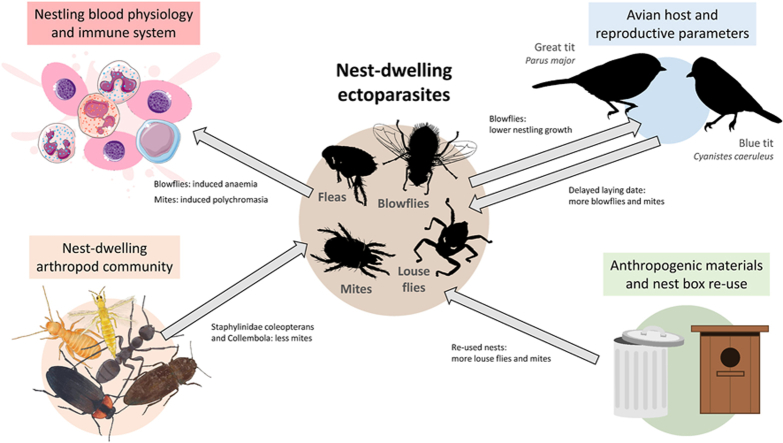

## 1 Introduction

Parasitism is an important limiting factor for breeding birds with strong impacts on their fitness (Newton, 1998). Hematophagous ectoparasites, such as fleas, bugs, lice, mites, ticks, louse flies and blowflies (Boyd, 1951), inhabit the nest during birds' breeding period and feed on nestlings' and parents' blood and interstitial tissue, and may transmit pathogens to their hosts. Among other effects, ectoparasites reduce nestlings' growth rate (Dudaniec and Kleindorfer, 2006; Dudek et al., 2021), body mass (Christe et al., 1996; Weddle, 2000) and mouth colouration (Dugas and Border, 2022), and increase nestling's immune system activity (Saino et al., 1998; Szép and Møller, 1999; Manzoli et al., 2018), vocal begging (Christe et al., 1996) and parental effort (Fitze et al., 2004; Knutie et al., 2016), as well as accelerate fledging (Møller, 1990; Chapman and George, 1991) and make parents abandon their nests (Brown and Brown, 1986). Ultimately, ectoparasites may impact host fitness by reducing short- and long-term survival of nestlings (Richner et al., 1993; Brown et al., 1995; Harriman and Alisauskas, 2010; Knutie et al., 2016; Manzoli et al., 2018), and lifetime reproductive success of parents (Fitze et al., 2004).

Because nestlings, but also parents (e.g. during incubation), are constrained to their nests, selection favours the evolution of a wide variety of anti-parasite behaviours to minimise the detrimental effects caused by ectoparasites (reviewed by Bush and Clayton, 2018). The selection of nest sites should be important, and their re-use by birds may increase the ectoparasite load (Mazgajski, 2007a; Tomás et al., 2007; González-Braojos et al., 2012; López-Arrabé et al., 2012). Consequently, breeding birds may avoid used and ectoparasite-infested nests from the previous breeding season (Merino and Potti, 1995a; Rytkönen et al., 1998).

Nests, especially those built in cavities, provide a suitable habitat for diverse arthropod communities (Di Iorio et al., 2008; Jaworski et al., 2022), in addition to ectoparasites. The type of cavity and the materials that parents choose to build their nests may alter the nest microhabitat, including temperature and humidity (Maziarz et al., 2017; Sudyka et al., 2022), and, consequently, the nest-dwelling arthropod community (Hanmer et al., 2017; Blunsden and Goodenough, 2023; Hanzelka et al., 2023; but see Moreno et al., 2002; Cantarero et al., 2013; Arce et al., 2018). This, in turn, may impact the ectoparasite community in the nest. For example, a high nest-dwelling arthropod diversity in the nest may be associated with a low abundance of fleas (Hanmer et al., 2017), which could benefit nestlings given the negative impacts of fleas on their health (Tripet and Richner, 1997; Pitala et al., 2009; Brommer et al., 2011). In fact, some ectoparasite predators, such as Histeridae or Staphylinidae coleopterans, prey on small parasites, including mites or fleas in the larval stage (Frank and Thomas, 2004; Capinera, 2008). Also, an increase in the number of nest-dwelling arthropods consuming nestlings' waste may contribute to nest sanitation and thus improve the breeding success (Krištofík et al., 2017).

Recently, the incorporation of anthropogenic materials in nests by birds (urban birds: Suárez-Rodríguez and Macías Garcia, 2014; Reynolds et al., 2016; Jagiello et al., 2022, 2023; seabirds: Battisti et al., 2019) has been suggested as a driver affecting the arthropod nest-dwelling communities and, indirectly, the ectoparasitic pressure (Hanmer et al., 2017). For example, an increase in anthropogenic materials in nests is associated with a lower nest-dwelling arthropod diversity, including arthropods that could act as ectoparasite predators (e.g. Histeridae and Staphylinidae coleopterans), but also to a higher abundance of ectoparasites (Hanmer et al., 2017; but see Reynolds et al., 2016). The anthropogenic materials, in contrast to natural materials, may provide a poorer environment for the development of nest-dwelling arthropods (Zettler et al., 2013; Parthasarathy et al., 2019).

Other factors that escape the control by breeding parents, such as abiotic factors related with environmental conditions experienced during the breeding season (e.g. temperature and rainfall; Merino and Potti, 1996), may play an important role in shaping the nest-dwelling arthropod communities. An increase in ambient temperature during the nestling stage may be positively related to prevalence of different flying ectoparasites (Eeva and Klemola, 2013). Nest microclimate, which is determined by environmental conditions (Berggren, 2005; Ardia, 2006), may also alter the nest-dwelling arthropod community, especially ectoparasite groups (Eeva et al., 1994; Heeb et al., 2000; Eeva and Klemola, 2013; Castaño-Vázquez et al., 2018, 2021), but also other arthropod groups such as coleopterans (García-Velasco et al., 2024). Therefore, the time of breeding is also important in shaping ectoparasite numbers in nests, and some studies have found an increase in nest-dwelling ectoparasites with laying or hatching date (Cantarero et al., 2013).

In this study, we aimed to compare the ectoparasitism levels of mites (obligatory and facultative parasitic mites), fleas and flies (*Protocalliphora azurea* blowflies and Hippoboscidae louse flies) between great tits (*Parus major*) and blue tits (*Cyanistes caeruleus*) breeding at Mata Nacional do Choupal, Coimbra (Central Western Portugal), during two consecutive breeding seasons. We evaluated how numbers of such ectoparasites varied along the breeding season in nests from these two tit species and how ectoparasites were associated with the breeding success and nestling physiology. We also explored the relationships between the use of anthropogenic materials in the nest and the nest-dwelling arthropod community, and between such non-parasite arthropod communities and ectoparasites. In addition, because estimating mite loads through direct counting after extraction of arthropods from the nest material is time-consuming and morose, we compared direct counts to two alternative field methods to estimate haematophagous mite infestation in order to assess their reliability.

## 2 Materials and methods

### 2.1 Study area and bird sampling

Data for this study was collected during the breeding seasons of blue tits and great tits nesting in nest boxes in Mata Nacional do Choupal (40°130N, 8°270W), Coimbra, Portugal, in 2021 and 2022. Nest boxes were cleaned prior to the start of each breeding season. This suburban mixed wood is composed by a wide variety of native and exotic tree species, including *Fraxinus angustifolia*, *Platanus* sp., *Acer negundo*, *Alnus glutinosa*, *Populus nigra*, *Laurus nobilis* and *Eucalyptus* sp. The understorey is composed mainly by *Tradescantia fluminencis*.

From the start of the breeding season, nest boxes were monitored to identify the species of the occupying breeding pair and obtain the laying date and basic breeding parameters for both tit species: clutch size, hatching date, brood size (nestlings counted at 3–5 days after hatching; hatching day = day 0), number of fledglings (counted at 14 days after hatching), and fledging success (number of nestlings that successfully fledged and left the nest in relation to the number of hatched nestlings). When nestlings were 14 days old, they were ringed, measured (tarsus length to the nearest 0.1 mm with a calliper), weighed (body mass to the nearest 0.1 g with a Pesola scale), and a blood sample was collected from the brachial vein. First, a drop of blood was used to make blood smears which were air-dried. Second, a portion of blood was maintained in a heparinised capillary tube in vertical position for 4 h at 4 °C to obtain the erythrocyte sedimentation rate (ESR, an indicator of acute infections and inflammation; Johnstone et al., 2017). Third, the remaining blood was maintained in another capillary tube and transported to the laboratory in a cool box. In the laboratory, in the same or following day, this last capillary tube (for great tits only) was centrifuged at 14,000 *g* for 10 min to measure the haematocrit, while the blood of the first capillary tube (once ESR was measured) was expelled into a microtube and frozen at −20 °C until haemoglobin measurement. The haemoglobin concentration was measured using the Drabkin method (Spinreact, Spain), according to manufacturer's instructions. Animal-origin haemoglobin (15 g/dL, Sigma) was used as the standard.

### 2.2 Nestling blood smears

The blood smears were fixed in methanol for 7 min and stained with Giemsa diluted 1:9 in distilled water for 45 min. Blood smears were observed under the microscope at 1000× magnification to obtain the white blood cell count (WBC), heterophil to lymphocyte ratio (H:L ratio), number of polychromatic erythrocytes (PCE), number of erythrocytic nuclear abnormalities (two groups: micronuclei, MNE; and other nuclear abnormalities, NAE), and nuclear abnormalities occurring in polychromatic erythrocytes (MNAEPCE). All these metrics were obtained per 10,000 erythrocytes following Garrido-Bautista et al. (2023). While the WBC is used to assess the status of the immune system and the presence of parasite infection (Owen et al., 2010), the H:L ratio is a generalist stress indicator, with high values indicating stressful situations due to the release of corticosterone into the blood stream (Davis et al., 2008). The simultaneous interpretation of WBC and H:L ratio may help to distinguish inflammation from disease and/or immunosuppression (Campbell, 2004). Erythrocytic nuclear abnormalities are more often used as indicators of genotoxicity caused by an increased oxidative damage produced by xenobiotics exposure. However, because oxidative stress can be a consequence of exposure to infection and other natural stressors, this metric could also be useful to evaluate trade-offs during exposure to other abiotic or biotic stressors (Bourgeon et al., 2012). Polychromasia increases due to the increase of PCE in the blood stream due to their higher rate of production (erythropoiesis), usually as a result of blood loss (either from injury or ectoparasitism) or erythrocyte destruction, thus reflecting a regenerative capacity of the individual (Campbell, 1995; Martinho, 2009).

Mean erythrocyte maturation index (EMI; Castro et al., 2018) has been reported to be a more sensitive metric to evaluate the bone marrow regenerative capacity than the counting of PCE (Maceda-Veiga et al., 2010). The EMI was obtained from photographing 10 microscopic fields per nestling at 1000× magnification. In each of these photographs, 13 erythrocytes were randomly measured along their shorter nuclear axis and longer cell axis in ImageJ version 1.52a (National Institutes of Health, Bethesda, MD, USA). The ratio of these measurements for each cell was used to calculate the mean EMI per nestling.

### 2.3 Field estimation of parasite abundance

Two field assessments of mite abundance were performed: the hand and bag methods. The hand method consisted of placing one hand inside the nest and touching the rim and middle of the nest cup for 1 min (Møller, 1990; Pryor and Casto, 2015). Then, mites present in the hand were counted (this procedure was done both when the nestlings were 6 and 14 days old). This method is based on mites being attracted to the hand's vibration and temperature (Owen and Mullens, 2004). Once the hand method was completed, we moved the nestlings from the nest into a white paper bag where they stayed for 5 min (bag method) (Dube et al., 2018). Then, the nestlings were transferred to a cloth bag and the mites present in the paper bag were counted and classified into the following classes: 0 = no mites, 1 = 1–10 mites, 2 = 11–30 mites, 3 = over 30 mites. We also registered the presence of louse flies (Hippoboscidae) flying off the nest or on nestling body parts when handling the nestlings. Louse flies could not be identified at the level of species given that their mobile behaviour precluded capturing them.

### 2.4 Nest collection and identification of nest-dwelling arthropods

Nests were visited every other day (maximum 3-days apart) during the estimated fledging period (18–22 days after hatching) to know the exact fledging date. When the nestlings left the nest, any nestling that died since ringing was accounted for and the nest material was collected into a plastic bag and then transported to the laboratory. Only successful nests (i.e. those in which at least one nestling fledged) were collected, as the arthropod community in nests that fail to produce fledglings changes rapidly due to the attraction of necrophagous insects, e.g. *Calliphora* larvae, *Dermestes* and *Silphidae* coleopterans (pers. obs.; Cosandey et al., 2021; García-Velasco et al., 2024), and would not accurately reflect the environment experienced by the nestlings during the nesting period. Arthropods from the nest material were extracted in a MacFadyen extractor during 48 h at 45 °C at the maximum of 3 days after collection. Extracted arthropods were maintained in 70 % ethanol until sorting and identification. Flea larvae and flea adults were analysed separately for the subsequent analyses. Nest material was visually sorted to collect *P. azurea* blowfly pupae, and then was dried for 48 h to obtain its dry weight to the nearest 0.01 g on an electronic balance.

Arthropods were classified in operational taxonomic units at the level of order, except ectoparasitic mites (classified at the level of genus), and Diptera and Coleoptera (at the level of family) to distinguish between ectoparasitic Diptera (blowflies) and predatory Coleoptera (Staphylinidae and Histeridae) (Frank and Thomas, 2004; Capinera, 2008; Hanmer et al., 2017). The identification was performed at the stereomicroscope using dichotomous taxonomic keys (Quigley and Madge, 1988). All arthropods were individually counted, except for mites, in which a subsample was used for extrapolation (see below). The Shannon diversity index of arthropods was calculated for each nest examined. For Diptera and Coleoptera, only families with over 10 % of representation of the total number of arthropods in any given nest were included in the diversity index. Staphylinidae and Histeridae families and facultative ectoparasitic mites were considered as potential predators given their feeding habits (Barker, 1968; Quigley and Madge, 1988; Frank and Thomas, 2004; Capinera, 2008), hence they were included as a group in some analyses (see below).

Mites were counted directly at the stereomicroscope or estimated when their abundance was very high, following Pacejka et al. (1996). Mites were divided into two groups according to their feeding habits: facultative parasitic mites for *Androlaelaps* sp. (Mesostigmata: Laelapidae), and obligatory parasitic mites, which include two genera: *Dermanyssus* sp. (Mesostigmata: Dermanyssidae) and *Ornithonyssus* sp. (Mesostigmata: Macronyssidae). To verify the classification of mites in each group carried out under the stereomicroscope, a proportion of mites was also identified under an optical microscope following specific literature (Till, 1963; Moss, 1978; Krantz and Walter, 2009; Radovsky, 2010). Both groups of mites were analysed separately in subsequent analyses.

### 2.5 Collection of nest anthropogenic materials

The sorting of anthropogenic materials was made visually by naked eye and, therefore, only macroscopic materials were considered. Anthropogenic materials were considered as any materials that do not exist naturally in the form or appearance they were found in the nests, i.e. anthropogenically-fabricated or processed materials, inclusively by techniques that make them more persistent, such as plastics and textiles. Single fibres were not included in the analyses. All materials that appeared to be non-natural were collected with the help of tweezers and placed in Petri dishes, duly identified with the nest identification and the date of collection. The materials were then observed under a stereomicroscope, with a magnification of 16.5×, to facilitate their classification. The relative quantity of anthropogenic materials per nest was estimated as the percentage of anthropogenic material weight in relation to the total nest dry weight.

### 2.6 Statistical analyses

The body condition index of nestlings was obtained from the residuals of the linear regression of body mass on tarsus length (log-transformed), separately by bird species (Labocha and Hayes, 2012). When comparing differences in ectoparasitic pressure between great tits and blue tits and along the breeding season, only first clutches were considered. Because nest dry weight was not associated with ectoparasite abundance (0.36 > *p* > 0.71, Spearman's correlation for all ectoparasite groups), this variable was not included in the statistical models. All subsequent analyses were performed in JMP Pro 17.0 software, except for the analyses of variation of ectoparasite abundance along the breeding season, which was conducted using the software R (The R Project for Statistical Computing; http://www.rproject.org). Means are expressed with standard error (SE), except stated otherwise.

#### 2.6.1 Arthropods in relation to tit species and nest characteristics

Nest material dry mass was compared between tit species with a Generalised Linear Model (GLM) fitted with normal distribution, with species and year as explanatory variables. To compare the prevalence of louse flies between great and blue tit nests, we used a logistic regression. The prevalence of louse fly adults was the dependent variable, while tit species (two levels: great and blue tit), year (two levels: 2020 and 2021) and the interaction between them were the explanatory variables. The interaction term was maintained when it improved the model performance (i.e. the interaction decreased the Akaike Information Criterion (AIC) over than 2). The comparison of ectoparasite abundance (total number of fleas, total number of blowflies and total number of obligatory or facultative ectoparasitic mites in infested and non-infested nests; Bush et al., 1997) between nests of the two tit species was performed using GLM, fitted with a Poisson distribution and linked to a log function. The abundance of ectoparasite groups was the dependent variable in separate models, while tit species, year and the interaction between them were the explanatory variables. The interaction term was only maintained if it improved the model performance (i.e. when the interaction decreased the AIC over than 2).

To evaluate the variation of ectoparasite abundance along the breeding season, we used GLM separately by tit species. The laying date (standardised as Julian date), year and their interaction (retained if it improved the model performance) were the explanatory variables, while the abundance of the ectoparasites were the dependent variables in separate models, fitted with tweedie distribution and log link function, except for flea abundance in blue tits, and obligatory and facultative parasitic mites in great tits, in which cases a negative binomial distribution was fitted and log link function. However, because we had only presence/absence data (i.e. prevalence) for louse flies, we ran a logistic regression with laying date as the explanatory variable and prevalence of louse fly adults as the dependent variable.

To examine the relationships between the relative quantity of anthropogenic materials in tit nests, the Shannon diversity index and the abundance of different arthropod groups (log-transformed; Supplementary Material 1), both ectoparasitic or not and including predators (see above), we used Spearman rank correlations separately by tit species. In the case of great tits, we used first and second broods pooled.

We also assessed the relationship between nest box re-use from one season to the next and the ectoparasite abundance. Although we cleaned up the nest boxes from one year to the next by removing old nest material, we did not sterilise them (eggs or other life stages of ectoparasites may overwinter in nest boxes). Because we installed nest boxes in spring 2020, in both 2021 and 2022 we still had nest boxes that were being used by tits for the first time. Therefore, we used chi-squared tests and Mann-Whitney tests to compare the prevalence (louse fly adults) and log-transformed abundance of ectoparasites (flea larvae and adult, blowfly larvae and pupae, obligatory and facultative parasitic mites), respectively, between nest boxes that were being used for the first time and nest boxes used in the previous breeding season. We used only first clutches from both tit species together.

#### 2.6.2 Ectoparasites and associations with nestling condition

The associations between the blowfly and both obligatoty and facultative ectoparasitic mites' abundance and the mean nestling morphometry per nest, mean nestling physiology per nest, and number of fledglings were tested with GLM separately per tit species. The dependent variables for separate models were nestling body mass, tarsus length, mean EMI, haematocrit, haemoglobin concentration, WBC, ESR, H:L ratio, PCE, MNE, NAE, and number of fledglings. Ectoparasite abundance was included as explanatory variable, along with year and its interaction with ectoparasite abundance. The interaction was only maintained if it improved the model performance. Brood size was included as a covariate. The GLMs were fitted with a normal distribution and linked to an identity function, except for the models for WBC, H:L ratio, PCE, MNE, NAE and number of fledglings, in which a Poisson distribution and log function were used. There was no data on ectoparasite abundance (louse flies) or they were present in very low intensity (fleas) to test associations between nestling condition and breeding success with the abundance of such parasites. Therefore, we compared nests with and without fleas or louse flies using separate GLMs with prevalence, year and its interaction, and brood size as explanatory variables, fitted either with normal distribution or Poisson distribution as above. The association of flea prevalence with haematocrit, haemoglobin concentration, WBC, H:L ratio, PCE, MNE and NAE in blue tit nests was not performed because there was only one blue tit nest without fleas for which we had such data. In all cases (i.e. models for nestling morphometry and physiology), we applied Bonferroni correction to control for Type I errors.

Both first and second broods were used to check for relationships with physiology and breeding success, arthropod communities and anthropogenic materials.

#### 2.6.3 Field methods to estimate mite abundance

We compared the abundance of mites (obligatory and facultative parasitic mites) between the classes of estimation of mite parasitism obtained by the hand and bag method with a Kruskal-Wallis test.

## 3 Results

### 3.1 Nest-dwelling ectoparasites in great and blue tit nests

We collected a total of 46 bluet tit (2021: 20, 2022: 26) and 67 great tit nests (2021: 32, 2022: 35). Nest material dry mass was significantly higher in blue tit than in great tit nests (great tit = 29.12 ± 1.18 g; blue tits = 32.88 ± 1.29 g; χ^2^_1, 85_ = 4.98, *p* = 0.03). Great tit nests had a higher prevalence of louse flies than blue tit nests, but only in 2021 (great tit: 9/22 (2021), 11/26 (2022); blue tit: 1/12 (2021), 10/20 (2022); species∗year: χ^2^_1, 67_ = 4.18, *p* = 0.040; Table 1).

**Table 1:** Prevalence and abundance of ectoparasites in blue (*Cyanistes caeruleus*) and great tit (*Parus major*) nests from Mata Nacional do Choupal, Portugal, in 2021 and 2022. Data from first broods.

	Blue tit				Great tit			
	2021		2022		2021		2022	
	mean ± SE	N	mean ± SE	N	mean ± SE	N	mean ± SE	N

Number of fleas (adults)	0.36 ± 0.28	11	0 ± 0	20	0.45 ± 0.14	22	0.57 ± 0.22	26
Number of fleas (larvae)	5.27 ± 5.27	11	66.05 ± 49.01	20	47.68 ± 19.37	22	23.23 ± 6.57	26
Total number of fleas	5.63 ± 5.53	11	66.05 ± 49.01	20	48.14 ± 19.38	22	23.81 ± 6.62	26
Number of blowflies (larvae)	1.09 ± 0.69	11	0.95 ± 0.64	20	0.54 ± 0.38	22	2.50 ± 2.23	26
Number of blowflies (pupae)	4.18 ± 2.22	11	2.5 ± 0.91	20	5.54 ± 2.23	22	0.73 ± 0.34	26
Total number of blowflies	5.27 ± 2.26	11	3.45 ± 1.00	20	6.09 ± 2.20	22	3.23 ± 1.04	26
Number of obligatory parasitic mites	5.54 ± 3.19	11	178.6 ± 78.49	20	66.62 ± 43.05	21	595.23 ± 229.28	26
Number of facultative parasitic mites	27.36 ± 12.28	11	53.05 ± 22.35	20	188.28 ± 46.62	21	147.69 ± 42.64	26

Prevalence of fleas (%)	18.2	11	25	20	63.6	22	69.2	26
Prevalence of obligatory parasitic mites (%)	90.9	11	100	20	85.7	21	96.2	26
Prevalence of facultative parasitic mites (%)	90.9	11	75	20	95.2	21	92.3	26
Prevalence of blowflies (%)	54.5	11	50	20	54.4	22	46.2	26
Prevalence louse flies (%)	8.30	12	45	22	40.9	22	42.3	26

Great tit nests had more obligatory parasitic mites (reference category = blue tit; estimate = 0.0013 ± 0.0004; χ^2^ = 12.84, *p* = 0.0003), and facultative parasitic mites (reference category = blue tit; estimate = 0.009 ± 0.001; χ^2^ = 60.96, *p* < 0.0001) than blue tit nests. Blue tit nests had significantly higher abundance of fleas than great tit nests in 2022 (estimate species∗year = 0.11 ± 0.02, χ^2^_1, 109_ = 96.93, *p* < 0.0001; Table 1). Blowfly abundance was only significantly affected by year, with higher values in 2021 (reference category = 2021; estimate = −0.33 ± 0.02, χ^2^_1, 109_ = 4.4, *p* < 0.04).

### 3.2 Anthropogenic materials, nest box re-use, and nest-dwelling arthropods

Anthropogenic materials were not correlated with the Shannon diversity index in either tit species (*p* > 0.05 in both cases), but were negatively correlated with the abundance of fleas (r_s_ = −0.41, *p* = 0.045) and positively correlated with the abundance of Histeridae (r_s_ = 0.44, *p* = 0.03) in great tit nests, and were associated with a lower abundance of booklice (Psocoptera) in blue tit nests (r_s_ = −0.62, *p* = 0.04). In great tit nests, the Shannon diversity index was positively correlated with the abundance of Staphylinidae (r_s_ = 0.26, *p* = 0.04) and negatively correlated with the abundance of obligatory parasitic mites (r_s_ = −0.29, *p* = 0.02). Whereas, in blue tit nests, the Shannon diversity index was positively correlated with the abundance of blowflies (r_s_ = 0.34, *p* = 0.02). In both great and blue tit nests, obligatory parasitic mite abundance was negatively correlated with the abundance of Staphylinidae (great tit: r_s_ = −0.45, *p* = 0.0001; blue tit: r_s_ = −0.37, *p* = 0.01) and Collembola (great tit: r_s_ = −0.32, *p* = 0.01; blue tit: r_s_ = −0.32, *p* = 0.03). In great tit nests only, obligatory parasitic mites were also less abundant with an increasing abundance of Diptera (r_s_ = −0.31, *p* = 0.01; Supplementary Material 2).

Re-used nest boxes showed a higher prevalence of louse flies (new nest boxes = 2/13; re-used nest boxes = 26/58; χ^2^_1, 71_ = 4.29, *p* = 0.04), and had a higher abundance of obligatory parasitic mites (new nest boxes = 91.0 ± 214.41, n = 13; re-used nest boxes = 337.3 ± 102.4, n = 57; one-tailed *t*_68_ = 1.86, *p* = 0.04) than nest boxes being used for the first time. No relationships between nest box re-use and abundance of blowflies, fleas or facultative parasitic mites were found (*p* > 0.05 in all cases).

### 3.3 Nest-dwelling ectoparasites, breeding performance and nestling morphology and physiology

**Fig. 1:**
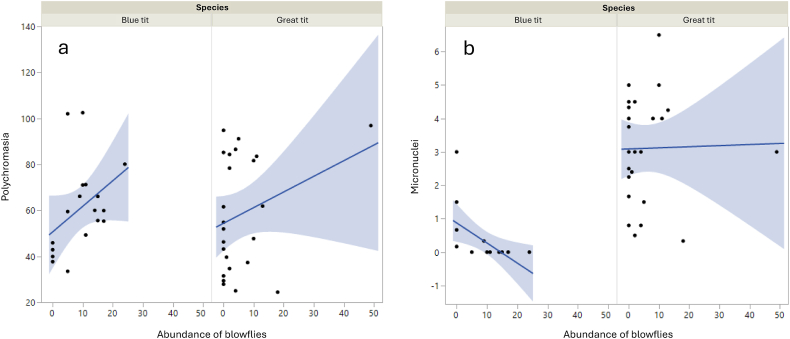
Relationship between the abundance of blowflies in nests and polychromasia (a) and number of micronuclei per 10,000 erythrocytes (b) in blue tit (*Cyanistes caeruleus*) and great tit (*Parus major*) nestlings. The lines are regression lines of predicted values and shades correspond to the CI-95 %. The scatterplots of raw data are also shown.

Blue and great tit breeding parameters and physiological parameters of their nestlings are presented in Supplementary Material 3. Blue tit nestlings from nests infested by louse flies had smaller tarsus than those from non-infected nests (reference category = louse flies; estimate = −0.29 ± 7.29; χ^2^_1, 43_ = 7.29, *p* = 0.007) and great tits from nests infested with fleas had higher polychromasia (PCE: estimate = 0.16 ± 0.03; χ^2^_1, 22_ = 33.3, *p* < 0.001 – significant after Bonferroni correction) and higher number of erythrocyte nuclear anomalies (NAE: estimate = 0.14 ± 0.05; χ^2^_1, 22_ = 7.02, *p* = 0.008). The higher the abundance of blowflies in great tit nests, the lower the nestling body mass of great tits (estimate = −0.047 ± 0.02; χ^2^_1, 65_ = 6.37, *p* = 0.02; Table 2; Supplemental Material 4) and tarsus length, although not significantly (estimate = −0.013 ± 0.007; χ^2^_1, 63_ = 3.54, *p* = 0.06; Table 2). Nestlings of both tit species showed lower levels of haemoglobin (blue tit: estimate = −1.12 ± 0.34; χ^2^_1, 39_ = 9.49, *p* = 0.002; great tit: estimate = −0.67 ± 0.21 χ^2^_1, 31_ = 9.16, *p* = 0.003) and higher polychromasia (blue tit: estimate = 0.02 ± 0.00, χ^2^_1, 16_ = 16.74, *p* < 0.0001; great tit: estimate = 0.01 ± 0.00, χ^2^_1, 22_ = 18.1, *p* < 0.0001; Fig. 1a) with increasing abundance of blowflies — these effects were significant after Bonferroni correction. In blue tit nests, the higher the abundance of blowflies, the lower the number of erythrocytic anomalies (both micronuclei and other anomalies; MNE: estimate = −0.44 ± 0.23, χ^2^_1, 16_ = 13.33, *p* = 0.0003; NAE: estimate = −0.92 ± 0.40; χ^2^_1, 16_ = 4.5, *p* = 0.03; Fig. 1b). The higher the abundance of blowflies, the higher the erythrocyte sedimentation rate in both tit species, but in the great tit this depended on year (blue tit, blowfly abundance: estimate = 0.28 ± 0.15, χ^2^_1, 43_ = 3.69, *p* = 0.05; great tit, year∗blowfly abundance: estimate = 0.11 ± 0.49, χ^2^_1, 57_ = 4.83, *p* = 0.03).

**Table 2:** Summary of the relationships between nest ectoparasites presence (fleas and louse flies) and abundance (blowflies and mites) and breeding performance or nestling morphology and physiology of great and blue tits. 0 no effect. – negative effect. + positive effect. n.a = not analysed. ∗*p* < 0.05, ∗∗*p* < 0.01, ∗∗∗*p* < 0.001. § significant effect after Bonferroni correction.

	Blue tit					Great tit				
	Louse flies	Blowflies	Obligatory parasitic mites	Facultative parasitic mites	Fleas	Louse flies	Blowflies	Obligatory parasitic mites	Facultative parasitic mites	Fleas
Number of fledglings	0	0	–∗	0	0	0	0	0	0	0

Body mass (g)	0	0	0	0	0	0	–∗	0	0	0
Tarsus length (mm)	–∗∗	0	0	–∗∗∗§	0	0	0	0	0	0

Mean erythrocyte maturation index	n.a	n.a	n.a	n.a	n.a	0	+∗∗	0	0	0
Haematocrit (%)	0	n.a.	n.a.	n.a.	n.a.	0	–	0	0	0
Erythrocyte sedimentation rate	0	+∗	0	0	0	0	+∗	0	+∗	0
Haemoglobin (g/dL)	0	–∗∗§	0	0	0	0	–∗∗§	0	0	0
White blood cell count	0	0	0	0	0	0	0	0	–∗	0
Heterophil/lymphocyte ratio	0	–∗	0	0	0	0	0	0	0	0
Erythrocyte micronuclei	0	–∗∗∗§	–∗∗	0	0	0	0	0	0	0
Other nuclear abnormalities	0	–∗	–∗∗	0	0	0	0	0	+∗∗	+∗∗
Polychromatic erythrocytes	0	+∗∗∗§	+∗∗∗§	0	0	0	+∗∗∗§	+∗∗∗§	0	+∗∗∗§

**Fig. 2:**
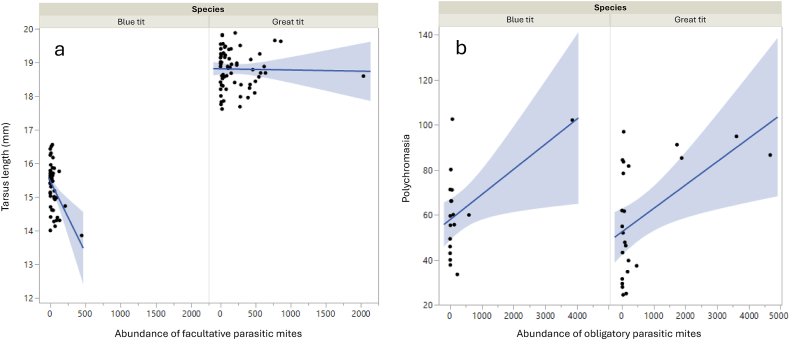
Relationships between the abundance of facultative parasitic mites in nests and tarsus length (a) and abundance of obligatory parasitic mites and polychromasia (b) in blue tit (*Cyanistes caeruleus*) and great tit (*Parus major*) nestlings. The lines are regression lines of predicted values and shades correspond to the CI-95 %. The scatterplots of raw data are also shown.

The abundance of facultative parasitic mites was negatively correlated with tarsus length in blue tit nestlings (estimate = −0.004 ± 0.001, χ^2^_1, 44_ = 10.95, *p* = 0.0009, significant after Bonferroni correction; Fig. 2a). In great tit nests, the abundance of facultative parasitic mites was negatively related with WBC (estimate = −0.002 ± 0.0001, χ^2^_1, 22_ = 5.92, *p* = 0.02), positively related to the number of erythrocytic nuclear anomalies in nestlings (NAE: estimate = 0.003 ± 0.0009, χ^2^_1, 22_ = 8.30, *p* = 0.004) and positively related with erythrocyte sedimentation rate, but only in 2021 (abundance of facultative parasitic mites∗year = 0.005 ± 0.002, χ^2^_1, 57_ = 4.11, *p* = 0.04). These relationships are summarised in Table 2.

In blue tit nests, the abundance of obligatory parasitic mites showed a negative association with the number of fledglings (estimate = −0.0004 ± 0.0002, χ^2^_1, 43_ = 4.95, *p* = 0.03). Also, the higher the abundance of obligatory parasitic mites in blue tit nests, the lower the number of erythrocytic anomalies (MNE: estimate = −0.06 ± 0.05, χ^2^_1, 16_ = 7.27, *p* = 0.007; NAE: estimate = −0.0002 ± 0.00009, χ^2^_1, 16_ = 7.01, *p* = 0.008). Nestlings of both tit species had elevated polychromasia in nests heavily infested by obligatory parasitic mites (great tit: estimate = 0.0001 ± 0.00002, χ^2^_1, 21_ = 53.4, *p* < 0.0001; blue tit: estimate = 0.0001 ± 0.00003, χ^2^_1, 16_ = 23.95, *p* < 0.0001; both significant after Bonferroni correction; Table 2; Fig. 2b).

### 3.4 Laying date and nest-dwelling ectoparasites

The laying date was significantly associated with the prevalence of louse flies in great tit nests and with the abundance of parasitic mites and blowflies in both great and blue tit nests (Supplementary Material 3). The abundance of blowflies increased along the breeding season in nests from both tit species (great tit: estimate = 0.050 ± 0.022; *p* = 0.024; blue tit: estimate = 0.072 ± 0.026; *p* = 0.007), and also the abundance of obligatory parasitic mites increased along the breeding season in blue tit nests (estimate = 0.067 ± 0.034; *p* = 0.049) and great tit nests (estimate = 0.075 ± 0.035; *p* = 0.033). The prevalence of louse flies decreased with the laying date in great tit nests (estimate = −0.026 ± 0.013; χ^2^_1, 66_ = 4.80, *p* = 0.03), but not in blue tit nests (*p* > 0.05). There were no significant relationships between the abundance of facultative parasitic mites with laying date in nests of any tit species (*p* > 0.05 in all cases).

### 3.5 Comparison of methods to estimate mite infestation

Mites were present in all 55 nests assessed for mite infestation after extraction in MacFadyen chamber: obligatory parasitic mites were present in 54 out of 55 nests, and facultative parasitic mites were present in 48 out of 55 nests. Using the MacFadyen extractor method, the mean intensity for obligatory parasitic mites was 178.6 ± 78.49 (n = 20) and 475.6 ± 178.6 (n = 34) in blue and great tit nests, respectively. The mean intensity for facultative parasitic mites was 70.73 ± 28.53 (n = 15) and 271.21 ± 73.02 (n = 33) in blue and great tit nests, respectively. However, using the hand method only 3 out of 55 nests had mites when nestlings were 6 days old, and only 1 out of 55 had mites when nestlings were 14 days old. Using the bag method, 15 out of 54 nests had mites when the nestlings were 14 days old (13 nests were classified as class 1 and 2 nests as class 2; no nest were classified as class 3).

The comparison of the results on mite infestation abundance using the hand method or extraction in MacFadyen chamber showed that for relatively low mite infestations (<500 obligatory parasitic mites), which were the majority of our cases (46/55), the hand method performed poorly with a false negative rate of 37/45 (82 %). Further results and discussion are presented in Supplementary Material 5.

## 4 Discussion

In this study we found that important drivers of ectoparasitic pressure for blue and great tits breeding in nest boxes include, besides the bird species, the laying date and the nest box re-use. We did not find support for the hypothesis that the incorporation of anthropogenic materials in nests was correlated to the nest-dwelling arthropod community or ectoparasite pressure, although relationships were found with some arthropod groups (e.g. Histeridae coleoptera and booklice). Regarding ectoparasites, we found that high nest infestations by blowflies were especially detrimental for both blue and great tit nestlings. Concretely, great tit nestlings reared in nests with high numbers of blowflies showed lower body mass and tarsus length, while nestlings of both tit species showed symptoms of regenerative anaemia (lower haemoglobin levels accompanied by higher numbers of polychromatic erythrocytes) with increasing numbers of blowflies in their nests. Also, blue tit nestlings from highly blowfly infested nests showed lower numbers of micronuclei anomalies in their erythrocytes. Similarly, both blue and great tit nestlings from highly infested nests with obligatory parasitic mites, and great tit nestlings from nests with high numbers of fleas, showed higher polychromasia. Lastly, high nest infestations with obligatory parasitic mites were marginally and negatively correlated with the number of fledged blue tits.

### 4.1 Ectoparasites in relation to host bird species

Great tit nests were generally more infested with both obligatory and facultative parasitic mites than blue tit nests. This pattern could be explained by differences in nest size or the exhibition of certain anti-parasitic behaviours between great and blue tits. Despite some within-species variation in nest size, blue tit nests tend to be heavier and taller than great tit nests (this study; Kaliński et al., 2014; Lambrechts et al., 2014, 2016). Given that nest size is generally positively correlated with the abundance of ectoparasites (Wittmann and Beason, 1992; Eeva et al., 1994; Kleindorfer and Dudaniec, 2009; but see Cantarero et al., 2013), blue tit nests were expected to harbour more parasitic mites than great tit nests. However, our results show the opposite pattern. Factors other than nest size in which the two tit species differ could intervene in determining mite development, such as the incorporation of green plant material by blue tits in their nests. The incorporation of aromatic plants in the nests is a behaviour rarely exhibited by great tits, but widely exhibited by blue tits to reduce ectoparasite and pathogen loads in the nest (Mennerat, 2008; Mennerat et al., 2009; Tomás et al., 2012). A recent study conducted in the same study area showed that mint, the most commonly used aromatic plant in nests by blue tits, had some repellent effects against fleas and parasitic mites (Garrido-Bautista et al., 2023), which could contribute to explain the results observed here. We also found that, in 2021, the prevalence of louse flies was higher in great tit nests than in blue tit nests. Since louse flies look actively for nests, intrinsic differences in host and host nest traits, such as scent, are relevant when finding suitable hosts. Hence, differences in nest composition might have an effect in attracting flies or repelling them. Additionally, larger brood size, host size and brood mass are all positively associated with louse fly occurrence and abundance (Roulin, 1998; Veiga and Valera, 2020), because larger hosts will increase the cues sought by the parasites, like carbon dioxide emissions or temperature (Andreasson et al., 2016). Therefore, larger hosts, like great tits, are expected to be preferred by louse flies than comparably smaller hosts, like blue tits.

### 4.2 Ectoparasites relationships with nestling physiology

Negative effects of blowfly parasitism on nestlings are widely known, especially in terms of reduced nestling growth rate and body size (Merino and Potti, 1995b, 1998; Hurtrez-Boussès et al., 1997; Morrison and Johnson, 2002; Bańbura et al., 2004; Thomas et al., 2007; Cantarero et al., 2013). In line with this, we found that the abundance of blowflies in nests tended to be negatively associated with great tit nestling growth: nestlings had smaller tarsi and lower body mass in blowfly heavily-infested nests. Impacts of ectoparasites, especially blowflies, on nestlings may differ depending on local habitat conditions (Musgrave et al., 2019; Maziarz et al., 2022; González-Bernardo et al., 2024), which may explain the observed variations in blowfly virulence. Also, the different capacity of parents to compensate for ectoparasitic costs by increasing the feeding effort (Hurtrez-Boussès et al., 1998; Bouslama et al., 2002; Simon et al., 2004) might mask some of the impacts of blowflies to nestlings.

Moreover, great and blue tit nestlings showed signs of anaemia (reduced haemoglobin concentration) when they were reared in blowfly heavily infested nests. This was accompanied by an elevated number of polychromatic erythrocytes which could indicate a capacity to recover from blood loss (i.e. regenerative anaemia; Martinho, 2009). Symptoms of anaemia in nestlings were already reported as a consequence of blowfly parasitism (Bouslama et al., 2001; O'Brien et al., 2001; Hannam, 2006; DeSimone et al., 2018; Grab et al., 2019), but few studies have focused on the capacity of the hosts to cope with such impacts by looking at further erythrocyte indices, such as red blood cell volumes and counts, as they mainly focused on haematocrit and haemoglobin concentration (Merino and Potti, 1998). The lower number of micronuclei in blue tit nestling in the presence of high blowfly parasitism could be related to blood loss and the decreased time for the erythrocytes to accumulate oxidative damage. We also observed that blowfly parasitism tended to be related to increased ESR in nestlings of both tit species, which is in accordance with results from a study conducted in blue tit nestlings in the study area (Garrido-Bautista et al., 2023). Overall, our results confirm that the haematophagous feeding activity of blowfly larvae impacts nestling physiology (i.e. provoking anaemia; Bouslama et al., 2001; O'Brien et al., 2001; Hannam, 2006; DeSimone et al., 2018; Grab et al., 2019), which probably translates into a decreased growth (i.e. reducing growth rate or final body size; Merino and Potti, 1995b, 1998; Hurtrez-Boussès et al., 1997; Morrison and Johnson, 2002; Bańbura et al., 2004; Thomas et al., 2007; Cantarero et al., 2013; Manzoli et al., 2018).

The impacts of haematophagous mites, such as *Dermanyssus gallinae* and *Ornithonyssus sylviarum*, on bird hosts are widely known in commercial poultry facilities (Mullens et al., 2009; Sigognault Flochlay et al., 2017), but the association between mite ectoparasitism and fitness of wild birds is less clear (Bauchau, 1997), although negative effects on haematocrit have been reported (Potti et al., 1999; Szabó et al., 2002; Pryor and Casto, 2015). In certain geographical regions, mites are considered one of the most virulent ectoparasites attacking breeding hole-nesting birds (López-Arrabé et al., 2012; and references therein). In our study, we detected a trend for lower fledging success in blue tits with increased infestation by obligatory parasitic mites. These results are in line with other studies conducted with tropical fowl mites (*Ornithonyssus bursa*) in barn swallows (*Hirundo rustica*) (Møller, 1990) and North Island robins (*Petroica longipes*) (Berggren, 2005). Contrastingly, tropical fowl mites had negligible effects on growth, physiology and survival of European starling (*Sturnus vulgaris*) nestlings (Powlesland, 1977). In our study, blue tits seemed to have not been able to cope with heavy mite infestations. In fact, significant associations of obligatory parasitic mites with nestling condition were mainly driven by the few cases of high mite infestations, in contrast to other studies in which mite infestations did not relate to nestling growth (Johnson and Albrecht, 1993; Pacejka et al., 1998) or fledging success (Johnson and Albrecht, 1993; Szabó et al., 2002). Nevertheless, although effects of mites on nestlings may not be detectable while in the nest, hosts might show deleterious effects of these ectoparasites in the post-fledging stage (Clayton and Tompkins, 1995). Nestlings reared in nests infested with obligatory parasitic mites showed higher polychromasia, indicating the capacity of nestlings to produce new erythrocytes in response to blood loss (Martinho, 2009), and a tendency for lower number of erythrocytic nuclear anomalies and micronuclei in the blue tit, as observed also when tit nestlings were parasitised by blowflies.

On the other hand, the presence of facultative parasitic mites *Androlaelaps* sp. seemed to be less harmful than obligatory parasitic mites, as only a negative relationship with tarsus length in blue tit nestlings was found. *Androlaelaps casalis* has been experimentally shown not to have detrimental effects on growth and short-term survival of nestlings (Pacejka et al., 1998), but may impact nestling body condition when found in high numbers in nests (Wolfs et al., 2012). However, the latter association must be viewed cautiously because it is influenced by the timing of nest mite abundance estimation: population of facultative parasitic mites increase up to fledging stage in detriment of obligatory parasitic mites, because facultative parasitic mites prey on obligatory parasitic mites (Wolfs et al., 2012). Our study highlights the importance of quantifying mite infestation by distinguishing between different mite groups according to their feeding habits when studying the costs imposed by mites on nestlings. Indeed, in a previous study conducted in the study area, we found that obligatory and facultative parasitic mites differ in their impacts on tit nestlings (Garrido-Bautista et al., 2023). Here, we support such differential effects of mites according to their trophic ecology.

Flea infestation has been previously shown to impact nestling condition and development in several ways, for example by reducing feather growth (Tripet and Richner, 1997; Brommer et al., 2011), immunocompetence (Pitala et al., 2009), haematocrit (Richner et al., 1993; Pitala et al., 2009; Brommer et al., 2011), growth (Pitala et al., 2009; Brommer et al., 2011) or the number of young fledged (Heeb et al., 2000). Contrary to expected, we did not detect any relationship between nest infestation by fleas and nestling growth in any tit species. This could be explained because the infestation levels achieved by fleas in the studied nests were of relatively low intensity compared to other geographical locations. We acknowledge that we did not count immobile phases of fleas (i.e. cocoons) in nests, so the extraction methodology used here may not be the most appropriate to assess flea infestation, as only mobile stages of arthropods are captured in the MacFadyen extractor. However, we found that in flea-infested nests great tits had higher polychromasia and tended to have lower erythrocytic nuclear anomalies, suggesting increased blood loss rate and higher rate of production of red blood cells. Louse flies were also shown to reduce nestling survival in some passerines (Eeva and Klemola, 2013), but we only found a tendency for a decreased tarsus growth in great tit nestlings in the presence of louse flies. Lastly, although we did not find any significant correlation among the abundances of different ectoparasite groups, we cannot exclude the possibility that ectoparasites may exhibit synergistic effects on nestlings when sharing the same nest (e.g. Merino and Potti, 1995b; Martínez-de la Puente et al., 2010). This, in turn, could be more likely to occur in years with poor environmental conditions (Dufva and Allander, 1996; but see Merino and Potti, 1995b). Also, given the correlative nature of our study, we can not confirm causality from the detected associations between ectoparasites and nestling health and breeding success because these relationships could be mediated by other factors not addressed here. For example, low-quality parents may have greater difficulties performing parental care, such as anti-ectoparasitic behaviours and/or feeding the nestlings properly to compensate for the negative effects of the parasites (e.g. Tripet and Richner, 1997; Bouslama et al., 2002; Cantarero et al., 2013). Likewise, low-quality nestlings may be more susceptible to being parasitised in the first place and provide less opposition to the proliferation of parasite populations (Beldomenico and Begon, 2010).

### 4.3 Nest-dwelling arthropod community and anthropogenic materials in the nest

Great tits from the study area incorporate more anthropogenic materials in their nests than blue tits (Girão et al., 2024). These differences in nest composition could potentially increase the intensity of infestation by some ectoparasites, such as fleas (Hanmer et al., 2017). We did not find support that the inclusion of anthropogenic materials in nests changed the arthropod community diversity (estimated as Shannon index), suggesting that levels of urbanisation may not have strong effects in modulating such nest communities (Baardsen et al., 2021). We neither observed that nests with a more diverse arthropod community had lower number of fleas (Hanmer et al., 2017). Still, our results suggest a direct and negative relationship between the relative quantity of anthropogenic materials and the abundance of fleas, although this association was found only for great tit nests. Contrastingly, the relationship between anthropogenic nest materials with ectoparasitic mites in great tit nests may be indirect through the modulation of the abundance of arthropod predators. In great tit nests, the relative quantity of anthropogenic materials was positively correlated with the abundance of Histeridae coleopterans, which are predators of small arthropods such as mites (Capinera, 2008). In nests of both tit species, obligatory parasitic mites were negatively correlated with the abundance of Staphylinidae coleopterans, which are also predators of small arthropods (Frank and Thomas, 2004). In partial accordance with Hanmer et al. (2017), we found that great tit nests with higher nest-dwelling arthropod diversity had more Staphylinidae coleopterans and less obligatory parasitic mites. However, our study differs from that of Hanmer et al. (2017) because we only considered broods that had not completely failed. This may have biased the results because the presence of dead nestlings and organic remnants in nests may alter the nest-dwelling arthropod community (Jaworski et al., 2022; García-Velasco et al., 2024).

### 4.4 Nest box re-use and ectoparasites

Nest box re-use between breeding seasons may increase the likelihood of ectoparasitic infestation (Tomás et al., 2007; González-Braojos et al., 2012; López-Arrabé et al., 2012; Zhang et al., 2023), especially for arthropod with resilient life stages that may overwinter in the nest boxes. For example, flea and mite populations are known to overwinter in nest boxes (Burtt Jr et al., 1991; Harper et al., 1992; but see Møller, 1990). We found that re-used nest boxes had indeed significantly higher number of obligatory parasitic mites than those being used for the first time, but also a higher probability of louse fly infestation. Experimental studies demonstrated that re-used nest boxes with old material harbour more ectoparasites, including blowflies, fleas and parasitic mites (Mazgajski, 2007b; Tomás et al., 2007; Blunsden and Goodenough, 2023), than fumigated and new nest boxes. Other studies showed differences in abundance between re-used and fumigated nests but depending on the ectoparasite group (López-Arrabé et al., 2012), suggesting that old nest material could attract some more ectoparasites than new and empty nests due to stronger odour cues. It should be noted that, in our study, nest material from the previous breeding season was removed. Still, it seems that the odour remaining in the used nest box might be attractive for louse flies. Our results thus confirmed early findings that the re-use of nest boxes determines the presence of ectoparasites, even if nest boxes have been cleaned of old nest material. Since mites may inhabit nest box crevices (Powlesland, 1977), fumigation is necessary to completely eliminate them from used nest boxes.

### 4.5 Breeding time and ectoparasites

We found that, in both tit species, broods that were reared later in the season harboured higher abundance of blowflies in their nests, as previously observed in other studies (Cantarero et al., 2013). These changes can be attributable to intra-seasonal climatic conditions, given the impact of nest microclimatic conditions on ectoparasite presence and development. For example, Heeb et al. (2000) experimentally demonstrated that the prevalence of blowflies increases as nest humidity decreases, which might explain why blowflies were more abundant in nests of late-breeding great and blue tits, because ambient temperature increased and humidity decreased with the advancing of the breeding season. Moreover, in a manipulative experiment, Dawson et al. (2005) showed that the number of blowflies in a nest varied in a curvilinear fashion with temperature, being highest at around 25 °C. In our study area, during the rearing period of first broods (April to May), the mean daily temperatures did not reach 25 °C and the maximum temperature was over 25 °C in less than half the days of the duration of first broods (data from: meteorological station of Escola Superior Agrária de Coimbra, located 1 km from Choupal). Regarding louse flies, some studies detected an increase in the abundance of louse flies with higher temperatures and in late broods (Eeva and Klemola, 2013). However, their study was performed in Finland, which might explain the discrepancy in relation to our finding that hyppoboscidae flies prevalence decreased with laying date.

Parasitic mite populations tend to increase as the breeding season progresses (Berggren, 2005), due to their short life cycles of few days (Sikes and Chamberlain, 1954) or weeks (Powlesland, 1977). Our findings pointed to such positive association of laying date with the number of obligatory parasitic mites in nests of both tit species. The life cycle, abundance and survival of mites are affected by temperature and humidity (Barker, 1968; Chen and Mullens, 2008; Castaño-Vázquez et al., 2018). Still, a possible explanation for the differential effect of seasonality on each mite group might be linked to inter-species differences in optimal climatic conditions and sensitivity (Barker, 1967). Indeed, population dynamics of mesostigmatid mites differ, even between closely related species, when exposed to the same abiotic factors (Guzmán et al., 2016).

## 5 Conclusions

In this study, we covered a large spectrum of nest-dwelling ectoparasites —including blowflies, fleas, obligatory and facultative parasitic mites and louse flies—and evaluated the potential drivers and consequences of their parasitisation levels in tit nests. We found that both nest box selection for reproduction and breeding time are important in determining nest-dwelling ectoparasite pressure, especially for blowflies and mites, while the amount of anthropogenic materials parents include in their nests did not influence infestation levels. Nest infestation by ectoparasites, especially blowflies and obligatory parasitic mites, had negative consequences for tit nestlings in terms of decreased growth and altered blood physiology, since nestlings showed symptoms of anaemia. Because we included in our analyses only nests that have not failed completely, at most we may have underestimated the effects of ectoparasites on breeding success and nestling physiology, and failed to find potential relationships between such ectoparasites and other arthropod taxa (e.g. *Dermestidae* necrophorous coleopterans; García-Velasco et al., 2024). In this sense, while most studies have only focused on the effects of ectoparasites on fledging success, morphometric measures, or at most on haemoglobin and haematocrit, we here encourage the use of other blood physiological metrics often ignored as health indicators. Our results showed that polychromasia evaluation and assessment of erythrocyte nuclear abnormalities are useful to evaluate regeneration rate of erythrocytes and if the host is able to cope with actual infestation levels.

## CRediT authorship contribution statement

**Sofía I. Arce:** Writing – review & editing, Writing – original draft, Investigation. **Jorge Garrido-Bautista:** Writing – review & editing, Writing – original draft, Investigation, Formal analysis. **Catarina G. Cascão:** Writing – review & editing, Investigation. **Inês S.C. Vilhena:** Investigation. **José Manuel Arjona:** Investigation. **Ana Rita Cabral:** Investigation. **Fábio Marengo:** Investigation. **Joana Girão:** Investigation. **Gregorio Moreno-Rueda:** Writing – review & editing, Resources. **Jaime A. Ramos:** Writing – review & editing, Resources, Conceptualization. **Ana Cláudia Norte:** Writing – review & editing, Writing – original draft, Formal analysis, Conceptualization.

## Availability of data and material

The data are freely available in the Supplementary Material of this article.

## Ethics statement

All applicable institutional and/or national guidelines for the care and use of animals were followed.

## Funding and logistic support

This study benefitted from funding provided by 10.13039/501100019370Foundation for Science and Technology, I. P (FCT) to 10.13039/501100019243MARE (UIDB/MAR/04292/2020; https://doi.org/10.54499/UIDB/04292/2020 and UIDP/04292/2020; https://doi.org/10.54499/UIDP/04292/2020) and the Associate Laboratory ARNET (LA/P/0069/2020; https://doi.org/10.54499/LA/P/0069/2020). Ana Cláudia Norte was supported by the transitory norm contract DL57/2016/CP1370/CT89. Sofía Irene Arce was supported by ‘Programa de financiamiento parcial para estadías en el exterior para becarios postdoctorales’ from Argentinian National Scientific and Technical Research Council and ‘Becas de movilidad con perspectiva de género’ from Production, Science and Technology Ministry of Santa Fe Province, Argentina. Jorge Garrido-Bautista was supported by a FPU pre-doctoral contract from the Spanish Ministry of Education (FPU18/03034) and by a mobility grant from ‘Asociación Universitaria Iberoamericana de Posgrado’ (AUIP).

## Declaration of competing interest

The authors declare they have no competing interests.
