# Supplementary material for: Drivers and consequences of nest ectoparasite pressure in tit nestlings
Source: Int J Parasitol Parasites Wildl. 2025 Apr 25;27:101075. doi: 10.1016/j.ijppaw.2025.101075 (PMC12861676; doi:10.1016/j.ijppaw.2025.101075)
Supplement: Multimedia component 1 [file mmc1.docx]

**Sup Mat 1.** Abundance of arthropods (excluding avian ectoparasites) in blue (*Cyanistes caeruleus*) and great tit (*Parus major*) nests from Mata Nacional do Choupal, Portugal, in 2021 and 2022. Data from first and second broods.

|  | Blue tit | | | | Great tit | | | |
| --- | --- | --- | --- | --- | --- | --- | --- | --- |
|  | 2021 | | 2022 | | 2021 | | 2022 | |
|  | Mean ± SE | N | Mean ± SE | N | Mean ± SE | N | Mean ±SE | N |
| **Arachnida** |  |  |  |  |  |  |  |  |
| Araneae | 0.15 ± 0.12 | 26 | 0.10 ± 0.07 | 20 | 0 ± 0 | 32 | 0.03 ± 0.03 | 35 |
| **Collembola** | 48.83 ± 8.52 | 23 | 14.50 ± 4.46 | 20 | 52.06 ± 14.37 | 32 | 6.65 ± 2.09 | 34 |
| **Insecta** |  |  |  |  |  |  |  |  |
| Coleoptera |  |  |  |  |  |  |  |  |
| Dermestidae | 0 ± 0 | 26 | 1.35 ± 0.90 | 20 | 2.25 ± 1.57 | 32 | 3.91 ± 2.07 | 35 |
| Histeridae | 0 ± 0 | 26 | 0.80 ± 0.80 | 20 | 0.13 ± 0.06 | 32 | 0 ± 0 | 35 |
| Staphilinidae | 22.08 ± 11.42 | 26 | 6.05 ± 3.81 | 20 | 134.16 ± 35.45 | 32 | 42.43 ± 23.12 | 35 |
| other Coleoptera | 1 ± 0.27 | 26 | 0.90 ± 0.45 | 20 | 2.81 ± 1.28 | 32 | 0.66 ± 0.31 | 35 |
| Dermaptera | 0.04 ± 0.04 | 26 | 0 ± 0 | 20 | 0.06 ± 0.06 | 32 | 0 ± 0 | 35 |
| Diptera  Muscidae | 0.77 ± 0.77 | 26 | 0 ± 0 | 20 | 0 ± 0 | 32 | 1.09 ± 0.95 | 35 |
| other Diptera | 1.08 ± 0.51 | 26 | 1.65 ± 1.55 | 20 | 1.19 ± 0.58 | 32 | 0.17 ± 0.14 | 35 |
| Hymenoptera |  |  |  |  |  |  |  |  |
| Formicidae | 4.23 ± 2.14 | 26 | 1.95 ± 1.12 | 20 | 3.00 ± 0.86 | 32 | 1.86 ± 0.84 | 35 |
| other Hymenoptera | 0.19 ± 0.10 | 26 | 1.55 ± 1.45 | 20 | 0.41 ± 0.15 | 32 | 0.63 ± 0.25 | 35 |
| Lepidoptera | 0.62 ± 0.16 | 26 | 0 ± 0 | 20 | 0 ± 0 | 32 | 0 ± 0 | 35 |
| Psocoptera (book lice) | 7.78 ± 1.93 | 23 | 19.70 ± 5.83 | 20 | 2.72 ± 0.80 | 32 | 12.74 ± 4.67 | 34 |
| Thysanoptera | 0.15 ± 0.09 | 26 | 0 ± 0 | 20 | 0 ± 0 | 32 | 0 ± 0 | 35 |
| **Malacostraca** |  |  |  |  |  |  |  |  |
| Isopoda | 0.00 ± 0.00 | 26 | 0 ± 0 | 20 | 0.41 ± 0.41 | 32 | 0.46 ± 0.40 | 35 |

**Sup Mat 2.** Spearman correlations (rs; P) between the abundance of nest dwelling arthropod groups and ectoparasites (log transformed), anthropogenic materials and Shannon diversity index. Shaded area – great tit; white area - blue tit;

|  | % anthropogenic material | Shannon Diversity Index | Fleas | Protocalliphora | Obligatory parasitic mites | Facultative parasitic mites | Predators | Collembola | Psocoptera | Staphylinidae | Histeridae | Dermestidae | Other Coleoptera | Diptera | Formicidae |
| --- | --- | --- | --- | --- | --- | --- | --- | --- | --- | --- | --- | --- | --- | --- | --- |
| % anthropogenic material | 1 | -0.54; 0.09 | -0.40; 0.22 | 0.43; 0.18 | 0.27; 0.42 | -0.23; 0.49 | -0.19; 0.58 | 0.39; 0.23 | -0.62; 0.04 | -0.26; 0.44 |  |  | 0.25; 0.46 | -0.34; 0.31 | 0.36; 0.27 |
| Shannon diversity index | 0.24; 0.27 | 1 | 0.09; 0.55 | 0.34; 0.02 | -0.26; 0.09 | 0.28; 0.06 | 0.25; 0.10 | 0.18; 0.25 | 0.19; 0.21 | 0.21; 0.16 | 0.14; 0.35 | 0.21; 0.15 | 0.12; 0.41 | 0.15; 0.32 | 0.13; 0.40 |
| Fleas | -0.41; 0.045 | 0.14; 0.26 | 1 | -0.11; 0.45 | 0.03; 0.84 | -0.09; 0.56 | -0.15; 0.32 | -0.05; 0.74 | 0.12; 0.46 | -0.12; 0.43 | -0.06; 0.68 | 0.13; 0.38 | -0.07; 0.64 | -0.18; 0.24 | 0.01; 0. 95 |
| Protocalliphora | 0.22; 0.31 | 0.14; 0.28 | -0.24; 0.05 | 1 | 0.006; 0.97 | 0.006; 0.97 | -0.02; 0.87 | 0.20; 0.19 | -0.27; 0.08 | -0.02; 0.89 | -0.18; 0.27 | -0.03: 0.84 | 0.13; 0.39 | 0.00; 0.98 | 0.18; 0.23 |
| Obligatory parasitic mites | 0.41; 0.05 | -0.28; 0.02 | -0.00; 0.978 | 0.14; 0.25 | 1 | 0.06; 0.71 | -0.03; 0.86 | -0.32; 0.034 | 0.12; 0.43 | -0.37; 0.01 | -0.03; 0.80 | 0.06; 0.68 | -0.07; 0.63 | -0.19; 0.23 | 0.03; 0.85 |
| Facultative parasitic mites | -0.07; 0.75 | -0.11;0.40 | -0.26; 0.03 | 0.10; 0.44 | -.015; 0.23 | 1 | 0.86; <0.0001 | 0.05; 0.76 | -0.03; 0.86 | 0.18; 0.22 | -0.08; 0.58 | 0.00; 0.97 | 0.02; 0.87 | 0.25; 0.10 | 0.15; 0.31 |
| Predators | -0.40; 0.05 | -0.08; 0.52 | -0.04; 0.72 | 0.13; 0.31 | -0.33; 0.008 | 0.79; <0.0001 | 1 | 0.15; 0.35 | -0.06; 0.71 | 0.45; 0.002 | 0.22; 0.13 | -0.05; 0.75 | 0.02; 0.91 | 0.48; <0.0007 | 0.08; 0.60 |
| Collembola | -0.03; 0.89 | 0.24; 0.05 | -0.11; 0.36 | 0.12; 0.33 | -0.32; 0.01 | 0.05; 0.68 | 0.18; 0.14 | 1 | -0.01: 0.95 | 0.28; 0.07 | 0.012; 0.93 | -0.07; 0.64 | 0.17; 0.28 | 0.03; 0.84 | 0.36; 0.02 |
| Psocoptera | 0.29; 0.17 | 0.16; 0.22 | 0.06; 0.63 | 0.08; 0.54 | -0.02; 0.86 | -0.20; 0.12 | -0.24; 0.06 | -0.02; 0.86 | 1 | -0.11; 0.45 | -0.15; 0.33 | 0.30; 0.05 | 0.02; 0.90 | 0.00; 0.98 | 0.07; 0.67 |
| Staphylinidae | -0.30; 0.15 | 0.26; 0.04 | 0.23; 0.06 | -0.10; 0.44 | -0.45; 0.0001 | 0.10; 0.40 | 0.49; <0.0001 | 0.36; 0.003 | -0.14; 0.25 | 1 | 0.24; 0.11 | -0.16; 0.28 | 0.22; 0.15 | 0.57; <0.001 | -0.12; 0.42 |
| Histeridae | 0.44; 0.003 | 0.23; 0.07 | -0.03; 0.82 | -0.02; 0.89 | -0.03; 0.83 | -0.08; 0.55 | -0.09; 0.48 | 0.23; 0.07 | 0.05; 0.66 | 0.04; 0.78 | 1 | -0.04; 0.80 | 0.29; 0.05 | 0.31; 0.03 | -0.10; 0.52 |
| Dermestidae | 0.02; 0.94 | 0.00; 0.98 | -0.22; 0.08 | 0.07; 0.59 | 0.15; 0.23 | -0.06; 0.61 | -0.19; 0.12 | -0.16; 0.21 | 0.05; 0.66 | -0.23; 0.06 | -0.12; 0.32 | 1 | 0.14; 0.34 | -0.11; 0.46 | 0.01; 0.96 |
| Other Coleoptera | -0.08; 0.71 | 0.18; 0.14 | 0.10; 0.42 | -0.01; 0.92 | -0.15; 0.23 | 0.05; 0.70 | 0.09; 0.46 | 0.12; 0.32 | -0.11; 0.38 | 0.11; 0.38 | 0.03; 0.80 | -0.04; 0.77 | 1 | 0.19; 0.18 | 0.01; 0.96 |
| Diptera | -0.35; 0.10 | 0.00; 0.99 | 0.04; 0.75 | 0.04; 0.76 | -0.31; 0.01 | 0.07; 0.59 | 0.38; 0.002 | 0.24; 0.05 | -0.24; 0.06 | 0.49; <0.0001 | -0.11; 0.40 | -0.10; 0.41 | 0.23; 0.06 | 1 | -0.11; 0.45 |
| Formicidae | 0.31; 0.14 | 0.05; 0.67 | -0.27; 0.03 | -0.10; 0.42 | -0.11; 0.39 | 0.02; 0.86 | -0.00; 0.96 | 0.40; 0.0008 | 0.04; 0.75 | 0.03; 0.84 | 0.19; 0.13 | -0.09; 0.46 | -0.03; 0.48 | -0.21; 0.08 | 1 |

**Sup Mat 3.** Breeding, morphological and physiological parameters of blue and great tits from Mata Nacional do Choupal, in 2020 and 2021. Based on mean brood values of first and second broods.

|  | Blue tits | | | | Great tits | | | |
| --- | --- | --- | --- | --- | --- | --- | --- | --- |
|  | 2021 |  | 2022 |  | 2021 |  | 2022 |  |
|  | mean ± SD | N | mean ± SD | N | mean ± SD | N | mean ± SD | N |
| Julian lay date | 124.12 ± 4.01 | 26 | 103.15 ± 1.75 | 20 | 117.06 ± 4.41 | 33 | 112.26 ± 3.72 | 35 |
| % anthropogenic material | 2.18 ± 0.44 | 11 |  | 0 | 9.57 ± 1.29 | 24 |  | 0 |
| Brood size | 4.50 ± 0.32 | 26 | 5.75 ± 0.35 | 20 | 4.45 ± 0.18 | 33 | 5 ± 0.21 | 35 |
| N fledgelings | 3.11 ± 0.30 | 26 | 4.5 ± 0.30 | 20 | 3.79 ± 0.27 | 33 | 4.37 ± 0.20 | 35 |
| Fledging success | 0.745 ± 0.06 | 26 | 0.800 ± 0.046 | 20 | 0.866 ± 0.038 | 33 | 0.883 ± 0.029 | 35 |
| Body mass (g) | 8.94 ± 0.23 | 26 | 9.36 ± 0.17 | 20 | 16.07 ± 0.28 | 33 | 16.06 ± 0.26 | 35 |
| Tarsus length (mm) | 15.29 ± 0.14 | 26 | 15.27 ± 0.15 | 20 | 18.80 ± 0.08 | 32 | 18.79 ± 0.11 | 34 |
| Body condition | 0.082 ± 0.14 | 26 | 0.022 ± 0.144 | 20 | 0.018 ± 0.205 | 32 | 0.070 ± 0.172 | 34 |
| Mean erythrocyte maturation index |  |  |  |  |  |  | 0.24 ± 0.00 | 24 |
| Haematocrit (%) |  |  |  |  |  |  | 42.04 ± 0.57 | 31 |
| Erythrocyte sedimentation rate | 13.51 ± 1.66 | 25 | 10.11 ± 0.93 | 20 | 11.90 ± 0.94 | 29 | 9.33 ± 0.52 | 33 |
| Haemoglobin (g/dL) | 86.30± 4.22 | 21 | 94.91 ± 2.84 | 20 |  | 0 | 104.51 ± 2.42 | 33 |
| White blood cell count | 22.43 ± 1.96 | 18 |  | 0 |  | 0 | 23.50 ± 1.60 | 24 |
| Heterophil: lymphocyte ratio | 0.52 ± 0.14 | 18 |  | 0 |  | 0 | 0.70 ± 0.10 | 24 |
| Erythrocyte micronuclei | 0.31 ± 0.18 | 18 |  | 0 |  | 0 | 3.11 ± 0.33 | 24 |
| Other nuclear abnormalities | 17.54 ± 3.20 | 18 |  | 0 |  | 0 | 15.19 ± 1.52 | 24 |
| Polychromatic erythrocytes | 61.04 ± 4.62 | 18 |  | 0 |  | 0 | 58.34 ± 5.10 | 24 |
| Nuclear abnormalities in polychromatic erythrocytes | 2.49 ± 1.16 | 18 |  | 0 |  | 0 | 0.34 ± 0.12 | 24 |

**Sup Mat 4.** Breeding, morphological and physiological parameters of infested and non-infested broods of great and blue tits breeding at Mata Nacional do Choupal, Coimbra, Portugal in 2021 and 2022. Data based on mean (± SE) brood values of first and second broods.

|  | | Obligatory parasitic mites | | | | | | | | | | | | | | | | | | | | | Facultative parasitic mites | | | | | | | | | | | | | | | | | | |  | |  | |  | |  | |  |
| --- | --- | --- | --- | --- | --- | --- | --- | --- | --- | --- | --- | --- | --- | --- | --- | --- | --- | --- | --- | --- | --- | --- | --- | --- | --- | --- | --- | --- | --- | --- | --- | --- | --- | --- | --- | --- | --- | --- | --- | --- | --- | --- | --- | --- | --- | --- | --- | --- | --- | --- |
|  | | Blue tit | | | | | Great tit | | | | | | | | | | | | | | | | Blue tit | | | | | | | | | Great tit | | | | | | | | | |  | |  | |  | |  | |  |
|  | | Absent | | | | | | Present | Absent | | | | | Present | | | | | | | | Absent | | | | | | Present | | | | | Absent | | | Present | | | | |  |  | |  | |  | |  | |  |
| N fledglings | | 0.00 | | | | 1 | | 3.60 ± 0.27 | 45 | | | 4.25 ± 1.44 | | | 4 | | 4.08 ± 0.16 | | | | | 63 | 4.10 ± 0.59 | | | | | 10 | 3.36 ± 0.31 | | | | 36 | 5 ± 0.81 | | | 3 | 4.05 ± 0.18 | | | 64 |  | |  | |  | |  | |  |
|  | |  | | | |  | |  |  | | |  | | |  | |  | | | | |  |  | | | | |  |  | | | |  |  | | |  |  | | |  |  | |  | |  | |  | |  |
| Body mass (g) | | 7.64 | | | | 1 | | 9.15 ± 0.15 | 45 | | | 15.98 ± 0.49 | | | 4 | | 16.06 ± 0.20 | | | | | 63 | 9.24 ± 0.33 | | | | | 10 | 9.09 ± 0.17 | | | | 36 | 16.26 ± 1.05 | | | 3 | 16.05 ± 0.20 | | | 64 |  | |  | |  | |  | |  |
| Tarsus length (mm) | | 14.13 | | | | 1 | | 15.31 ± 0.10 | 45 | | | 18.60 ± 0.16 | | | 4 | | 18.81 ± 0.07 | | | | | 61 | 15.64 ± 0.21 | | | | | 10 | 15.18 ± 0.11 | | | | 36 | 18.81 ± 0.21 | | | 3 | 18.80 ± 0.07 | | | 62 |  | |  | |  | |  | |  |
|  | |  | | | |  | |  |  | | |  | | |  | |  | | | | |  |  | | | | |  |  | | | |  |  | | |  |  | | |  |  | |  | |  | |  | |  |
| Mean erythrocyte maturation index | | na | | | | 0 | | na | 0 | | | na | | | 0 | | 0.24 ± 0.00 | | | | | 24 | na | | | | | 0 | na | | | | 0 | 0.25 | | | 1 | 0.24 ± 0.00 | | | 23 |  | |  | |  | |  | |  |
| Haematocrit (%) | | na | | | | 0 | | na | 0 | | | 45.31 | | | 1 | | 41.93 ± 0.58 | | | | | 30 | na | | | | | 0 | na | | | | 0 | 38.95 ± 4.15 | | | 2 | 42.25 ± 0.55 | | | 29 |  | |  | |  | |  | |  |
| Erythrocyte sedimentation rate | | 8.13 | | | | 1 | | 12.09 ± 1.05 | 44 | | | 12.19 ± 2.04 | | | 4 | | 10.35 ± 0.57 | | | | | 57 | 14.19 ± 2.18 | | | | | 10 | 11.40 ± 1.17 | | | | 35 | 11.26 ± 2.54 | | | 3 | 10.43 ± 0.56 | | | 58 |  | |  | |  | |  | |  |
| Haemoglobin (g/dL) | | 116.25 | | | | 1 | | 89.86 ± 2.61 | 40 | | | 90.83 | | | 1 | | 104.93 ± 2.46 | | | | | 32 | 92.0 1 ± 5.38 | | | | | 10 | 90.02 ± 3.06 | | | | 31 | 111.71 ± 1.93 | | | 2 | 104.04 ±2.55 | | | 31 |  | |  | |  | |  | |  |
| White blood cell count | | 19.00 | | | | 1 | | 22.64 ± 2.07 | 17 | | | na | | | 0 | | 23.50 ± 1.60 | | | | | 24 | 23.14 ± 4.28 | | | | | 4 | 22.23 ± 2.29 | | | | 14 | 35.2 | | | 1 | 22.99 ± 1.59 | | | 23 |  | |  | |  | |  | |  |
| Heterophil: lymphocyte ratio | | 1.80 | | | | 1 | | 0.44 ± 0.12 | 17 | | | na | | | 0 | | 0.70 ± 0.10 | | | | | 24 | 0.46 ± 0.30 | | | | | 4 | 0.53 ± 0.16 | | | | 14 | 0.64 | | | 1 | 0.70 ± 0.10 | | | 23 |  | |  | |  | |  | |  |
| Erythrocyte micronuclei | | 0.29 | | | | 1 | | 0.29 ± 0.19 | 17 | | | na | | | 0 | | 3.11 ± 0.33 | | | | | 24 | 0.04 ± 0.39 | | | | | 4 | 0.39 ± 0.20 | | | | 14 | 2.4 | | | 1 | 3.14 ± 0.34 | | | 23 |  | |  | |  | |  | |  |
| Other nuclear abnormalities | | 16.00 | | | | 1 | | 17.63 ± 3.40 | 17 | | | na | | | 0 | | 15.19 ± 1.52 | | | | | 24 | 15.77 ± 6.99 | | | | | 4 | 18.04 ± 3.73 | | | | 14 | 20.8 | | | 1 | 14.95 ± 1.57 | | | 23 |  | |  | |  | |  | |  |
| Polychromatic erythrocytes | | 45.9 | | | | 1 | | 61.94 ± 4.81 | 17 | | | na | | | 0 | | 58.33 ± 5.10 | | | | | 24 | 60.00 ± 10.01 | | | | | 4 | 61.34 ± 5.40 | | | | 14 | 39.66 | | | 1 | 59.15 ± 5.25 | | | 23 |  | |  | |  | |  | |  |
|  |  | | | | | | | | | | | | | | | | | | |  | | | | | | | | | | | | | | | | | | |  |  |  |  | |  | |  | |  | |  |
|  |  | | | | | | | | | | | | | | | | | | |  | | | | | | | | | | | | | | | | | | |  |  |  |  | |  | |  | |  | |  |
|  | Fleas | | | | | | | | | | | | | | | | | | | Louse flies | | | | | | | | | | | | | | | | | | |  |  |  |  | |  | |  | |  | |  |
|  | Blue tit | | | | | Great tit | | | | | | | | | | | | | | Blue tit | | | | | | | | | | Great tit | | | | | | | | | |  |  |  | |  | |  | |  | |  |
|  | Absent | | | | | | | Present | Absent | | | | | Present | | | | | | | Absent | | | | | | Present | | | | | Absent | | | Present | | | | | | |  | | | | |  | |  |  |
| N fledglings | 3.41 ± 30 | | | | 39 | | | 4.41 ± 0.70 | 7 | 3.9 ± 0.29 | | | 30 | | | 4.24 ± 0.21 | | | | | | | 37 | 3.25 ± 0.33 | | | | 32 | 4.23 ± 0.51 | | | | 13 | 4.09 ± 0.20 | | 45 | | 4.09 ± 0.31 | | | | | 23 | |  | | | |  |  |
|  | | |  |  |  |  |  |  |  |  |  |  |  |  |  |  |  |  |  |  |  |  |  |  |  |  |  |  |  |  |  |  |  |  |  |  |  |  |  |  |  |  |  |  |  |  |  |  |  |  |
| Body mass (g) | 9.17 ± 0.16 | | | | 39 | | | 8.86 ± 0.38 | 7 | 15.94 ± 0.30 | | | 30 | | | 16.27± 0.23 | | | | | | | 37 | 9.02 ± 0.18 | | | | 32 | 9.45 ± 0.25 | | | | 13 | 15.85 ± 0.24 | | 45 | | 16.48 ± 0.31 | | | | | 23 | |  | | | |  |  |
| Tarsus length (mm) | 15.33 ± 0.11 | | | | 39 | | | 15.04 ± 0.22 | 7 | 18.73 ± 0.10 | | | 29 | | | 18.86 ± 0.09 | | | | | | | 36 | 15.13 ± 0.11 | | | | 32 | 15.71 ± 0.12 | | | | 13 | 18.76 ± 0.07 | | 45 | | 18.88 ± 0.15 | | | | | 21 | |  |  |  |  |  |  |
|  | | |  |  |  |  |  |  |  |  |  |  |  |  |  |  |  |  |  |  |  |  |  |  |  |  |  |  |  |  |  |  |  |  |  |  |  |  |  |  |  |  |  |  |  |  |  |  |  |  |
| Mean erythrocyte maturation index | na | | | | 0 | | | na | 0 | 0.25 ± 0.00 | | | 12 | | | 0.24 ± 0.00 | | | | | | | 12 | na | | | | 0 | na | | | | 0 | 0.25 ± 0.00 | | 13 | | 0.24 ± 0.00 | | | | | 11 | |  | | | |  |  |
| Haematocrit (%) | na | | | | 0 | | | na | 0 | 42.44 ± 0.83 | | | 15 | | | 41.66 ± 0.80 | | | | | | | 16 | na | | | | 0 | na | | | | 0 | 41.99 ± 0.81 | | 18 | | 42.10 ± 0.79 | | | | | 13 | |  |  |  |  |  |  |
| Erythrocyte sedimentation rate | 12.34 ± 1.12 | | | | 39 | | | 9.81 ± 1.39 | 6 | 10.94 ± 0.79 | | | 27 | | | 9.74 ± 0.59 | | | | | | | 34 | 12.07 ± 1.27 | | | | 31 | 11.66 ± 1.58 | | | | 13 | 10.86 ± 0.77 | | 39 | | 9.97 ± 0.66 | | | | | 23 | |  |  |  |  |  |  |
| Haemoglobin (g/dL) | 90.70 ± 2.88 | | | | 35 | | | 89.34 ± 3.55 | 6 | 105.08 ± 4.03 | | | 15 | | | 104.03 ± 3.00 | | | | | | | 18 | 88.64 ± 3.27 | | | | 27 | 93.56 ± 4.72 | | | | 13 | 106.59 ± 3.12 | | 20 | | 101.30 ± 3.81 | | | | | 13 | |  |  |  |  |  |  |
| White blood cell count | 22.43 ± 2.08 | | | | 18 | | | 22.5 | 1 | 25.10 ± 1.77 | | | 12 | | | 21.90 ± 2.67 | | | | | | | 12 | 21.38 ± 2.16 | | | | 14 | 22.83 ± 1.09 | | | | 3 | 23.20 ± 2.46 | | 13 | | 23.87 ± 2.06 | | | | | 11 | |  |  |  |  |  |  |
| Heterophil: lymphocyte ratio | 0.54 ± 0.14 | | | | 17 | | | 0.16 | 1 | 0.93 ± 0.13 | | | 12 | | | 0.48 ± 0.12 | | | | | | | 12 | 0.55 ± 0.17 | | | | 14 | 0.38 ± 0.36 | | | | 3 | 0.79 ± 0.14 | | 13 | | 0.60 ± 0.14 | | | | | 11 | |  |  |  |  |  |  |
| Erythrocyte micronuclei | 0.33 ± 0.19 | | | | 17 | | | 0 | 1 | 3.46 ± 0.56 | | | 12 | | | 2.76 ± 0.34 | | | | | | | 12 | 0.39 ± 0.21 | | | | 14 | 0.06 ± 0.46 | | | | 3 | 3.30 ± 0.46 | | 13 | | 2.88 ± 0.48 | | | | | 11 | |  |  |  |  |  |  |
| Other nuclear abnormalities | 18.06 ± 3.35 | | | | 17 | | | 8.75 | 1 | 17.31 ± 2.25 | | | 12 | | | 13.08 ± 1.95 | | | | | | | 12 | 18.33 ± 3.84 | | | | 14 | 14.36 ± 8.3 | | | | 3 | 15.58 ± 2.25 | | 13 | | 14.73 ± 2.10 | | | | | 11 | |  |  |  |  |  |  |
| Polychromatic erythrocytes | 61.74 ± 4.85 | | | | 17 | | | 49.3 | 1 | 67.31 ± 6.85 | | | 12 | | | 49.36 ± 6.85 | | | | | | | 12 | 57.05 ±4.64 | | | | 14 | 65.87 ±10.02 | | | | 3 | 57.84 ±7.08 | | 13 | | 58.92 ± 7.69 | | | | | 11 | |  | | | |  |  |
|  | Blowflies | | | | | | | | | | | | | | | | | | | | | | | | | | | | | | | |  |  |  |  |  |  |  |  |  |  | |  | |  | |  | |  |
|  | Blue tit | | | | | | | | | | | | | | | | | | Great tit | | | | | | | | | | | | | |  |  |  |  |  |  |  |  |  |  | |  | |  | |  | |  |
|  | Absent | | | | | | | | | | Present | | | | | | | | Absent | | | | | | | Present | | | | | | |  |  |  |  |  |  |  |  |  |  | |  | |  | |  | |  |
| N fledglings | 3.82 ± 0.45 | | | | | | | 17 | | | 3.34 ± 0.35 | | | | | | | 29 | 4.21 ± 0.21 | | | | | | 28 | 4 ± 0.26 | | | | | 39 | |  |  |  |  |  |  |  |  |  |  | |  | |  | |  | |  |
|  |  | | | | | | |  | | |  | | | | | | |  |  | | | | | |  |  | | | | |  | |  |  |  |  |  |  |  |  |  |  | |  | |  | |  | |  |
| Body mass (g) | 9.44 ± 0.24 | | | | | | | 17 | | | 8.93 ± 0.19 | | | | | | | 29 | 16.62 ± 0.24 | | | | | | 28 | 15.77 ± 0.26 | | | | | 39 | |  |  |  |  |  |  |  |  |  |  | |  | |  | |  | |  |
| Tarsus length (mm) | 15.53 ± 0.16 | | | | | | | 17 | | | 15.14 ± 0.13 | | | | | | | 29 | 18.89 ± 0.11 | | | | | | 27 | 18.74 ± 0.09 | | | | | 38 | |  |  |  |  |  |  |  |  |  |  | |  | |  | |  | |  |
|  |  | | | | | | |  | | |  | | | | | | |  |  | | | | | |  |  | | | | |  | |  |  |  |  |  |  |  |  |  |  | |  | |  | |  | |  |
| Mean erythrocyte maturation index | na | | | | | | | 0 | | | na | | | | | | | 0 | 0.24 ± 0.00 | | | | | | 10 | 0.25 ± 0.00 | | | | | 14 | |  |  |  |  |  |  |  |  |  |  | |  | |  | |  | |  |
| Haematocrit (%) | na | | | | | | | 0 | | | na | | | | | | | 0 | 43.13 ± 0.89 | | | | | | 14 | 41.14 ± 0.68 | | | | | 17 | |  |  |  |  |  |  |  |  |  |  | |  | |  | |  | |  |
| Erythrocyte sedimentation rate | 9.80 ± 1.65 | | | | | | | 17 | | | 13.34 ± 1.28 | | | | | | | 28 | 9.96 ± 0.68 | | | | | | 26 | 10.50 ± 0.67 | | | | | 35 | |  |  |  |  |  |  |  |  |  |  | |  | |  | |  | |  |
| Haemoglobin (g/dL) | 98.54 ± 3.92 | | | | | | | 16 | | | 85.36 ± 3.14 | | | | | | | 25 | 108.73 ± 2.96 | | | | | | 15 | 100.98 ± 3.55 | | | | | 18 | |  |  |  |  |  |  |  |  |  |  | |  | |  | |  | |  |
| White blood cell count | 20.38 ± 4.25 | | | | | | | 4 | | | 23.02 ± 2.27 | | | | | | | 14 | 22.61 ± 2.37 | | | | | | 10 | 24.14 ± 2.22 | | | | | 14 | |  |  |  |  |  |  |  |  |  |  | |  | |  | |  | |  |
| Heterophil: lymphocyte ratio | 0.97 ± 0.27 | | | | | | | 4 | | | 0.39 ± 0.15 | | | | | | | 14 | 0.52 ± 0.10 | | | | | | 10 | 0.84 ± 0.15 | | | | | 14 | |  |  |  |  |  |  |  |  |  |  | |  | |  | |  | |  |
| Erythrocyte micronuclei | 1.33 ± 0.27 | | | | | | | 4 | | | 0.02 ± 0.15 | | | | | | | 14 | 3.18 ± 0.43 | | | | | | 10 | 3.06 ± 0.48 | | | | | 14 | |  |  |  |  |  |  |  |  |  |  | |  | |  | |  | |  |
| Other nuclear abnormalities | 33.08 ± 5.44 | | | | | | | 4 | | | 13.10 ± 2.91 | | | | | | | 14 | 14.13 ± 2.07 | | | | | | 10 | 15.95 ± 2.19 | | | | | 14 | |  |  |  |  |  |  |  |  |  |  | |  | |  | |  | |  |
| Polychromatic erythrocytes | 41.61 ± 8.47 | | | | | | | 4 | | | 66.60 ± 4.53 | | | | | | | 14 | 52.67 ± 7.19 | | | | | | 10 | 62.38 ± 7.08 | | | | | 14 | |  |  |  |  |  |  |  |  |  |  | |  | |  | |  | |  |

**Sup. Mat 5**. ***Field estimation of parasite abundance***

Although there were significant differences between nests estimated in the field using the bag method as classes 0, 1 or 2 in the number of obligatory parasitic mites (χ^2^_2,52_ =12.24, *p* = 0.002) estimated using the McFayden extractor method, there were no significant differences in the number of facultative parasitic mites between different classes (χ^2^_2,52_ = 1.12, *p* = 0.60). Also, there were 38 nests classified as without mites by the bag method which indeed had obligatory parasitic mites (mean intensity = 92.0 ± 131.5) when using the McFayden extractor method. The nests classified as class 1 using the bag method had 827.69 ± 1307 obligatory parasitic mites, and those classified as class 2 had 2,674 ± 1,326 obligatory parasitic mites, according to the McFadyen extraction method.

To obtain accurate estimates of abundance of nest-dwelling mesostigmatid mites associated to birds, such as *Androlaelaps* sp., *Ornithonyssus* sp. and *Dermanyssus* sp. (Proctor and Owens, 2000), one needs to sort and examine nest material. The direct counting of mites extracted from the nest material is, however, very time consuming and relatively difficult when identifying mite immature stages, hence potential reliable alternative methods would be welcome. In addition to this, non-destructive techniques to quantify mite abundance while nestlings still remain in the nest have the advantage of taking sequential samples of mite load along the nestlings’ development, which would allow to pinpoint causal associations between parasites and hosts (Beldomenico and Begon, 2010). We tested alternative field methods of mite infestation intensity focused on blood feeding mites dwelling on the nestling host skin, which would still be a relevant proxy of mite ectoparasitic pressure. However, it did not seem to be robust for low infestation levels (which were the majority of our observations) because we detected 82% of false negatives. Previous studies found a correlation between the actual count of parasitic mites extracted from the nest material and mite field counts which included mites counted on the hand after placing it in the nest, mites observed on the nestlings, and mites in the container where nestlings were held (Møller, 1990; Merino and Potti, 1996; Dube et al., 2018). Given that the negative impacts we detected on the hosts were mainly driven by high infestation levels, while low infestation levels did not seem to cause major threats to the health and survival of nestlings, this could be an alternative and faster assessment of mite abundance to consider in studies focused on associations of mites and birds, while recognising the loss of sensitivity of this technique.

**References**

Beldomenico, P.M., Begon, M., 2010. Disease spread, susceptibility and infection intensity: vicious circles? Trends. Ecol. Evol., 25(1), 21-27. doi: 10.1016/j.tree.2009.06.015

Dube, W.C., Hund, A.K., Turbek, S.P., Safran, R.J., 2018. Microclimate and host body condition influence mite population growth in a wild bird-ectoparasite system. Int. J. Parasitol.: Parasites and Wildl., 7(3), 301-308. doi: 10.1016/j.ijppaw.2018.07.007

Merino, S., Potti, J., 1996. Weather dependent effects of nest ectoparasites on their bird hosts. Ecography, 19(2), 107-113. doi: 10.1111/j.1600-0587.1996.tb00161.x

Møller, A.P., 1990. Effects of parasitism by a haematophagous mite on reproduction in the barn swallow. Ecology*,* 71(6), 2345-2357. doi: 10.2307/1938645

Proctor, H., Owens, I., 2000. Mites and birds: diversity, parasitism and coevolution. Trends Ecol. Evol., 15(9), 358-364. doi: 10.1016/S0169-5347(00)01924-8
